# The fractal geometry of nutrient exchange surfaces does not provide an explanation for 3/4-power metabolic scaling

**DOI:** 10.1186/1742-4682-2-30

**Published:** 2005-08-11

**Authors:** Page R Painter

**Affiliations:** 1 Office of Environmental Health Hazard Assessment, California Environmental Protection Agency, P. O. Box 4010, Sacramento, California 95812, USA

## Abstract

**Background:**

A prominent theoretical explanation for 3/4-power allometric scaling of metabolism proposes that the nutrient exchange surface of capillaries has properties of a space-filling fractal. The theory assumes that nutrient exchange surface area has a fractal dimension equal to or greater than 2 and less than or equal to 3 and that the volume filled by the exchange surface area has a fractal dimension equal to or greater than 3 and less than or equal to 4.

**Results:**

It is shown that contradicting predictions can be derived from the assumptions of the model. When errors in the model are corrected, it is shown to predict that metabolic rate is proportional to body mass (proportional scaling).

**Conclusion:**

The presence of space-filling fractal nutrient exchange surfaces does not provide a satisfactory explanation for 3/4-power metabolic rate scaling.

## Background

Physiological variables (*e.g*., cardiac output) or structural variables (*e.g*., pulmonary alveolar surface area) in mammals of mass *M *in many cases are closely approximated by an exponential function, *R *= *R*_1_*M*^*b*^, which is termed an allometric relationship [1,2]. A prominent example is Kleiber's law for scaling the basal metabolic rate (BMR) in mammals [3,4], *B *= *B*_1_*M*^3/4^, which is equivalent to scaling the specific BMR, *B/M*, proportionally to *M*^-1/4^.

In the report, "The Fourth Dimension of Life: Fractal Geometry and Allometric Scaling of Organisms," West, Brown and Enquist (WBE) [5] derive the 3/4-power law in part from the claim that mammalian distribution networks are "fractal like" and in part from the conjecture that natural selection has tended to maximize metabolic capacity "by maximizing the scaling of exchange surface areas" for the delivery of oxygen and nutrients to body tissues.

WBE derive an expression describing scaling of surface area for nutrient exchange by considering a scale transformation that increases the linear dimensions of arteries and other internal structures (with the exception of capillaries) by the factor *λ*. The dimensions of individual capillaries are assumed to be invariant. WBE express scaling of the total internal exchange area as

(1)

where ***a ***is the area following the transformation and ***a***_***r ***_is the area before. The authors describe the exponent ***2+ε***_***a ***_as the "fractal dimension of ***a***" to justify the restriction 0 ≤ ***ε***_***a ***_≤ 1 (Assumption 1). They justify the upper limit of ***ε***_***a ***_by stating that ***ε***_***a ***_= 1 "represents the maximum fracticality of a volume-filling structure in which the effective surface area scales like a conventional volume." This makes it clear that the structures in their model exist in 3-dimensional Euclidean space, E_3_.

Similarly, they express scaling of ***v***, the internal volume associated with ***a ***(and assumed to be proportional to body mass), as

(2)

where ***3+ε***_***v ***_is termed the "fractal dimension of ***v***" and ***ε***_***v ***_satisfies 0 ≤ ***ε***_***v ***_≤ *I *(Assumption 2). They then write ***v ***= ***al***, defining a new function ***l***, which is assumed to be an internal linear dimension other than that of capillaries. The scaling of ***l ***is described by the equation

(3)

where ***ε***_***l ***_is again a parameter that satisfies 0 ≤ ***ε***_***l ***_≤ 1 (Assumption 3). From the above equation for ***v***, it follows that

(4)

The last assumptions of the theory are that natural selection has tended to maximize metabolic capacity "by maximizing the scaling of exchange surface areas" (Assumption 4) and that BMR is proportional to ***a ***(Assumption 5). Maximization of ***a/v ***requires ***ε***_***a ***_= 1, and ***ε***_***l ***_= 0 (Result 1). Consequently, the fractal dimension of ***a ***is 3 (Result 2), and the fractal dimension of ***v ***is 4 (Result 3). Substitution of these values into Equations (2) and (4) followed by elimination of *λ *leads to

***a/v ***∝ ***v***^-1/4 ^           (5)

which is a form of Kleiber's law if Assumption 5 is true.

In a critical review of the WBE model, Dodds *et al*. [6] claim that "the bounds 0 ≤ ***ε***_***a***_, ***ε***_***v***_, ***ε***_***l ***_≤ 1 are overly restrictive." They analyze the example where 0 ≤ ***ε***_***a***_, ***ε***_***v ***_≤ 1, as in the WBE model, but where -1 ≤ ***ε***_***l ***_≤ 1. Optimization leads to the conclusion that the fractal dimension of ***l ***is 0, that the fractal dimension of both ***a ***and ***v ***is 3 and that exchange surface area ***a ***scales with volume. Consequently, ***a/v ***is constant in this example.

Agutter and Wheatley [7] also critically reviewed the WBE model, pointing out that the maximal metabolic rate (MMR) is plausibly limited by nutrient supply while the BMR is not limited by nutrient supply. Therefore, the model of WBE should predict the scaling of MMR. However, the scaling exponent for MMR appears to be different from 3/4. Weibel *et al*. [8] estimate this exponent to be 0.872 with a 95% confidence interval of (0.812 – 0.931).

While the issue raised by Agutter and Wheatley may not be resolvable using mathematical analysis, the issue raised by Dodds *et al*. is readily addressed by mathematical analysis. In the following, the theory of WBE is evaluated by first using a model of a 3-dimensional fractal-like network. Then the rigor of the arguments used in deriving the results of the theory is evaluated using properties of Hausdorff n-dimensional measure, the concept that is the basis for the general definition of fractal dimension.

## Results

**Figure 1:**
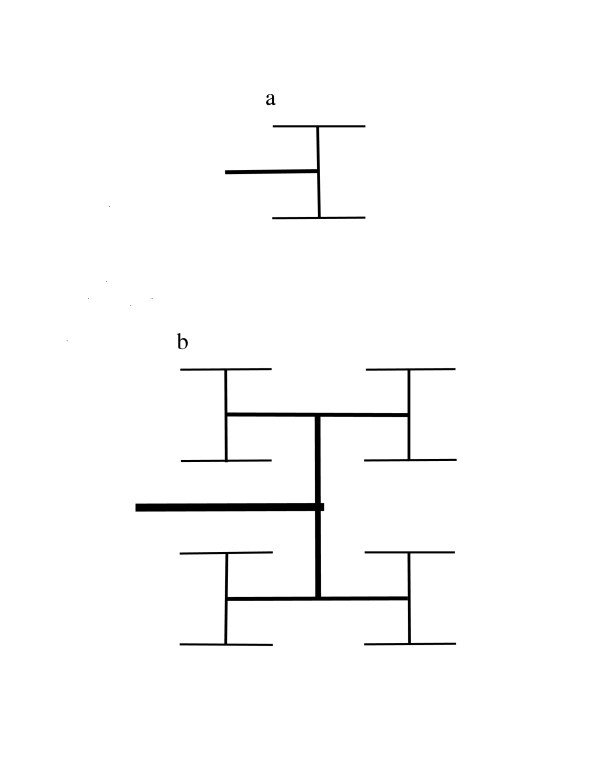
A 2-dimensional fractal-like, branching network model for an arterial tree. Blood enters the network through the structure represented as a thick horizontal line. Terminal arteries are represented by thin horizontal lines. a. A network that uniformly supplies a 2 × 2 area where the unit distance is the spacing between adjacent termini of small arteries. b. A network that uniformly supplies a 4 × 4 area.

**Figure 2:**
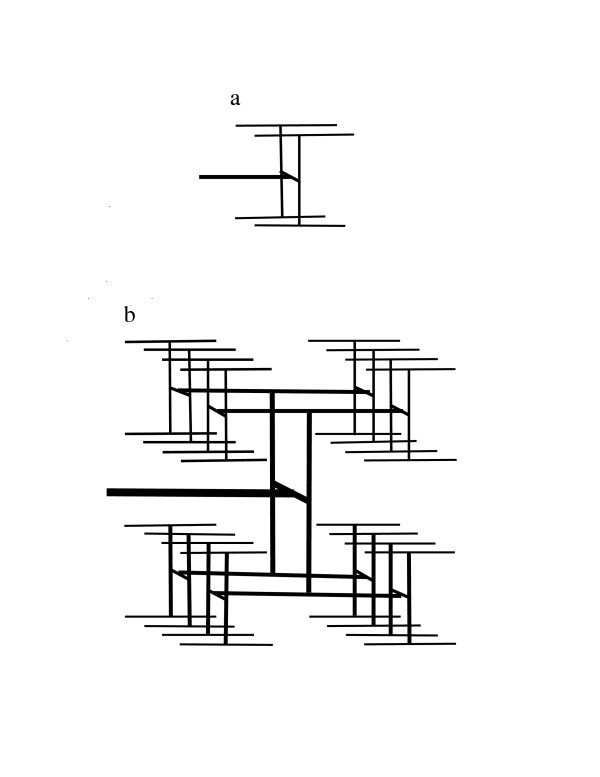
A 3-dimensional fractal-like, branching network model for an arterial tree. Blood enters the network through the structure represented as a thick horizontal line. Terminal arteries are represented by thin horizontal lines. a. A network that uniformly supplies a 2 × 2 × 2 volume where the unit distance is the spacing between adjacent termini of small arteries. b. A network that uniformly supplies a 4 × 4 × 4 volume.

If the argument used by WBE to "prove" 3/4-power scaling is valid, it should require 3/4-power scaling for a specific example of a fractal distribution network. Examples of the "fractal-like" arterial networks previously described by WBE [9] are shown in Figures 1 and 2. The supply network for a square starts with an H-shaped network that is connected to the nutrient source (Figure 1a). The network is extended by iteratively connecting each terminal site to an H-shaped structure that is one-half the size (in terms of linear dimension) of the structures added in the previous step (Figure 1b). For a network that supplies a cube, we start with two parallel H-shaped structures that are connected by a conduit. This structure, termed an H-H structure, is illustrated in Figure 2a. This network is extended by iterative additions of H-H structure of one-half the dimension of the previously added H-H structure. Each added structure is connected at its midpoint. Iterative addition of smaller and smaller H-shaped structures in Figure 1 gives the fractal lung model of Mandelbrot [10], and iterative addition of H-H structures gives a 3-dimensional fractal model. An infinite sequence of additions gives an area-filling network of fractal dimension 2 for the 2-dimensional network and a space-filling network of fractal dimension 3 for the 3-dimensional network. The 2-dimensional network in Figure 1 is equivalent to the fractal-like network illustrated in Figure 4 of Turcotte *et al*. [11], and the 3-dimensional network in Figure 2 is equivalent to the fractal-like network in Figure 7 of Turcotte *et al*.

We now compare the maximum nutrient exchange surface area for the network shown in Figure 2a with that of the network shown in Figure 2b. We assume that, for both networks, each terminus is connected to capillaries that have an associated fractal surface. Their "maximum fracticality" is the dimension 3, which corresponds to a space-filling surface. The measure of the exchange surface area is the volume of the space within V that is filled by the surface. This volume is assumed by WBE to be proportional to total body volume and to body mass. Therefore, we can write *A = cV*, where *c *≤ 1. Consequently, exchange surface area and metabolic rate scale proportionally to volume for the networks in this example. This in turn implies that *λ*^3 ^is proportional to *V*. However, WBE conclude that *V *is proportional to *λ*^4^. Consequently, there must be an error in their argument.

The source of the contradiction is Equation (4), which WBE justify by claiming "***v ***can always be expressed as ***v = al***, where ***l ***is some length characteristic of the internal structure of the organism." In conventional geometry, this assertion is correct for certain types of figures when area is defined as the cross-sectional area. For example, the volume of a right cylinder is equal to the area of its circular cross section multiplied by the length of the cylinder. The volume is not equal to the exterior surface area of the cylinder multiplied by its length. Unfortunately, WBE assume that in fractal geometry, unlike the arithmetic of conventional geometry, volume is exterior surface area multiplied by length. The assumption that fractal volume is equal to fractal cross-sectional area multiplied by length leads to the conclusion that volume scales as *λ*^3^. This is because a cross-section of a fractal in E_3 _is the intersection of a plane and the fractal, *i.e*., it is a set of points in 2-dimensional space, just as is the case for conventional geometric objects. With this correct calculation of the (maximum) dimension of a fractal object with surface area ***a ***and length ***l***, it follows that the metabolic rate is proportional to volume and body mass, as illustrated by the example in Figure 2.

## Discussion

At the heart of the argument of WBE is the hypothesis that a 3-dimensional object in E_3 _with volume V can be filled with a fractal surface to produce an object with fractal dimension 4. This hypothesis is false because the volume of an object has the same, finite value whether an object contains a space-filling fractal surface or not. Because the volume is finite (and not equal to 0), the Hausdorff dimension (which is a general definition of fractal dimension) must be equal to 3 [12]. If the presence of a space-filling surface within the object changes its fractal dimension to a value greater than 3, then the Hausdorff dimension is greater than 3 and the conventional volume is infinite. This contradiction in the WBE model is resolved by rejecting all assumptions that allow for the Hausdorff dimension of a mammalian body to be greater than 3, the maximum dimension of an object in E_3_. These are Equations (2), (3) and (4) and Assumptions (2) and (3). Equation (3) and Assumption (3) must be rejected because ***l ***is not a set of points in space and therefore has no fractal dimension. When these assumptions are removed, the maximum nutrient exchange surface area principle of WBE leads to the prediction that metabolic rate is directly proportional to body mass.

The assumption that relates exchange surface area to metabolic rate, Assumption 5, is a common assumption used to explain diffusion-limited nutrient uptake. It seems plausible for non-fractal exchange surfaces such as the walls of capillaries, which are approximately cylindrical. For such surfaces, area is proportional to Hausdorff 2-dimensional measure. Hausdorff 3-dimensional measure of such surfaces is 0. Therefore, metabolic rate must be proportional to 2-dimensional measure of the surface if the WBE formulation is to be biologically meaningful. However, when the capillary surface is extended to a space-filling fractal that scales as volume, Hausdorff 2-dimensional measure is infinite, but Hausdorff 3-dimensional measure is proportional to the volume filled by the fractal. Therefore, the Hausdorff 3-dimensional measure must be used to scale metabolic rate for space-filling surfaces if the formulation is to be biologically meaningful. While this dichotomy in the computation of rate-determining area is not a mathematical contradiction, it does result in losing the standard justification for Assumption 5, because nutrient diffusion rate and metabolic rate cannot be proportional to exchange surface area when the fractal dimension of exchange surface area is 3. Furthermore, Assumption 5 leads to the conclusion that all space-filling exchange surfaces filling the same volume *V *of a mammalian body must confer exactly the same metabolic rate on the organism. This seems peculiar because the connection of function with biological form appears to have been lost as a result of the application of WBE's maximization principle, Assumption 4.

As discussed in the background section, Dodds *et al*. [6] claim that the bounds on fractal dimensions in the WBE model are "too restrictive" and replace Assumption 3 by extending the allowable values of the fractal dimension of ***l ***to the interval [0, 2]. However, their criticism is not valid because the bounds on fractal dimensions in the WBE model are not too restrictive. They are not restrictive enough.

## Competing interests

The author(s) declare that they have no competing interests.
